# SAR image wave spectra to retrieve the thickness of grease-pancake sea ice using viscous wave propagation models

**DOI:** 10.1038/s41598-021-82228-x

**Published:** 2021-02-01

**Authors:** Giacomo De Carolis, Piero Olla, Francesca De Santi

**Affiliations:** 1 National Research Council of Italy (CNR), Institute for Electromagnetic Sensing of the Environment (IREA), Milan, 20133 Italy; 2 National Research Council of Italy (CNR), Institute of Atmospheric Science and Climate (ISAC), Cagliari, 09042 Italy; 3 National Institute for Nuclear Physics (INFN), Cagliari, 09042 Italy

**Keywords:** Fluid dynamics, Physical oceanography

## Abstract

Young sea ice composed of grease and pancake ice (GPI), as well as thin floes, considered to be the most common form of sea ice fringing Antarctica, is now becoming the “new normal” also in the Arctic. A study of the rheological properties of GPI is carried out by comparing the predictions of two viscous wave propagation models: the Keller model and the close-packing (CP) model, with the observed wave attenuation obtained by SAR image techniques. In order to fit observations, it is shown that describing GPI as a viscous medium requires the adoption of an ice viscosity which increases with the ice thickness. The consequences regarding the possibility of ice thickness retrieval from remote sensing data of wave attenuation are discussed. We provide examples of GPI thickness retrievals from a Sentinel-1 C band SAR image taken in the Beaufort Sea on 1 November 2015, and three CosmoSkyMed X band SAR images taken in the Weddell Sea on March 2019. The estimated GPI thicknesses are consistent with concurrent SMOS measurements and available local samplings.

## Introduction

Climate change has led in the last decade to a dramatic reduction in the extent and the thickness of sea ice in the Arctic region during the summer season. As a result, the marginal ice zone (MIZ), which is the dynamic transition region separating the ice pack from the open ocean, has become increasingly exposed to the wind and the wave actions^1,2^. In these conditions, water freezes to form a thin slurry of frazil ice, called grease ice, with thicknesses up to 10–30 cm depending on the windy conditions^3–5^. As the freezing process continues, it leads to a further stage of development of the grease ice layer, which consolidates into pancake-shaped ice bodies, called pancake ice^6,7^. Grease and pancakes ice (GPI) have always been relatively ubiquitous in the Antarctic MIZ^8^, and extensive GPI formation has recently been observed also in the western Arctic, as documented during the field campaign in fall 2015^9^.

Investigations to determine how an increase in GPI may affect the climate, possibly leading to local warming in the far north, are ongoing. These studies and their implementation in climate models clearly require some tools to monitor the GPI’s properties, especially as regards its thickness.

As the extent of GPI fields is controlled by the presence of waves that usually come from the open ocean, the relationship between wave attenuation rates and GPI rheology is a key factor to understand and model the evolution of the ice cover. Given the large scale separation between pancakes and the typical wavelength, a good approximation to describe the interaction with ocean waves is to represent GPI as a continuum^10,11^. Validation and calibration of any GPI wave model are tasks that cannot be entrusted to in situ activities, due to the vast extent of the GPI fields, their intrinsic dynamic nature, and the harsh environment where they develop. A systematic and extensive characterization of the spatio–temporal distribution of GPI can however be carried out through remote sensing by exploiting the capability of SAR imaging techniques to measure the full ocean wave directional spectrum^12^. By tracking the SAR observed ocean wave spectrum (or its peak, as done in pioneer studies such as^13^), from open sea throughout the GPI cover, it is indeed possible to measure wave dispersion and attenuation. A dedicated GPI wave model can then be used to relate the modifications in the wave propagation with GPI properties such as its thickness and mechanical properties.

The literature on the application of SAR spectral inversion techniques to GPI thickness retrieval dates back at least to 1997. In earlier papers^14–17^, the GPI thickness was estimated by a adopting a mass-loading scheme, in which GPI is represented as a continuum of non-interacting point-like mass loads on the sea surface^10^. However, the success of the approach was limited by the excessively high values of the thickness resulting from SAR inversion.

Later papers^18–20^ abandoned the mass-loading approach for the more realistic representation of GPI proposed by Keller^11^. The Keller’s model pictures the GPI layer as a viscous fluid of given thickness *h* and effective viscosity $\nu$ floating over an infinitely deep, inviscid water column. The GPI effective viscosity is an unknown parameter, with experimental evidence suggesting strong variability with respect to the composition of the sea ice matrix. As an example, wave buoy attenuation data collected in the Weddell Sea over GPI fields with pancakes thicknesses up to 50 cm, compared with the Keller’s models, revealed a variability of GPI effective viscosity values spanning the range $10^{-1}-10^{3}\,{\mathrm{m}}^2/{\mathrm{s}}$^21^, without a clear relationship with the GPI layer thickness.

Other viscous layer models have been proposed to describe wave propagation in ice/water systems. We can mention the two-layer viscous model (TLV), which assumes the water underneath the ice cover to have a finite viscosity, possibly due to turbulent effects^22^, and the viscoelastic model, which treats the ice layer as a viscoelastic medium^23^. All these generalizations of the Keller’s model, however, seem unable to significantly reduce the variability of the effective ice viscosity^24,25^.

We argue that at least part of the variability of the effective viscosity required by the Keller’s model to describe the properties of GPI, may stem from attempting to treat the ice layer as a homogeneous medium. The close-packing (CP) model^26^ makes a first attempt to explicitly take into account the inhomogeneity of GPI, by assuming that pancakes are confined to a fictitious layer of infinitesimal thickness, lying on top of the grease ice layer and modifying the wave stress at the upper surface. The CP model was tested on field data from the Arctic and the Antarctic, along with Keller’s and TLV models^25^. Fitting of the field data was possible with all the models considered, but only the CP model produced GPI effective viscosities in a range consistent with that of grease ice in laboratory experiments ($\approx 2.5{-}3 \times 10^{-2}\,{\mathrm{m}}^2/{\mathrm{s}}$)^27^.

The SAR inversion procedure consists of the following four steps:

- Estimation of the directional open ocean wave spectrum ${\tilde{S}}_w(\mathbf{k})$ at the boundary of the ice-field from the available SAR image, using the closed form expression of the non-linear ocean-to-SAR spectral form described in Hasselmann and Hasselmann (1991)^12^ and later modified for the SAR image cross-spectral transform^28^.
- Simulation of the ocean wave spectrum in sea ice $S_i(\mathbf{x},\mathbf{k};h,\nu )$. It is assumed that GPI cover alters the incoming ocean wave spectrum according to the following expression^19,29^:
  1 $$\begin{aligned} S_i(\mathbf{x},\mathbf{k};h,\nu )={{\tilde{S}}}_w(\mathbf{k})\exp [-2q(h,\nu )\Delta (\mathbf{e}_\mathbf{k},\mathbf{x})], \end{aligned}$$
  where $\Delta (\mathbf{e}_\mathbf{k},\mathbf{x})$ is the distance traveled from the ice edge to the position $\mathbf {x}$ by the wave of wavenumber $\mathbf {k}$ heading the direction $\mathbf{e}_\mathbf{k}$ and $q(h,\nu )$ is the wave damping predicted by the viscous wave propagation model considered.
- Transformation of the simulated wave spectrum to a SAR image spectrum $P_i(\tau ;h,\nu ;\mathbf{x},\mathbf{k}))$, as described in^12^,
- Comparison of the modeled and the observed SAR spectrum, ${\tilde{P}}_i(\tau ;\mathbf{x},\mathbf{k})$, through the following cost function
  2 $$\begin{aligned} \min _{\nu ,h}\ \Psi (\tau ,\mathbf{x};h,\nu )=\int [\mathfrak {R}(\tilde{P}_i(\tau ;\mathbf{x},\mathbf{k})-P_i(\tau ;h,\nu ;\mathbf{x},\mathbf{k}))]^2{\mathrm{d}}^2k, \end{aligned}$$
  where $\mathfrak {R}$ stands for the real part of the argument and $\tau$ the temporal separation between the SAR looks (in the cases in exam $\tau =0$).

The procedure is therefore characterized by the choice of the viscous wave propagation model to predict the wave damping. Predictions on $q(h,\nu )$ provided by the TLV and the viscoelastic models have been carried out elsewhere and showed not to differ significantly from the ones by the Keller’s model at a given GPI’s thickness and viscosity^24,25^. The present study is therefore limited to the Keller’s and the CP model. An outline of the models is presented in the “Methods” section.

In the former SAR inversion schemes, the couple $(h,\nu )$ minimizing the distance—parameterized by the cost function—between simulated and observed SAR image spectrum, was then assigned to the GPI portion traveled by the waves. However, we have observed that the intrinsic structure of GPI viscous wave models does not allow one to identify an absolute minimum of the cost function. Instead, deep valleys in the parameters’ space can be observed, which leads to important consequences on the procedure of ice thickness retrieval^25^. The valleys follow power-law curves $\nu \propto h^\alpha$, where $\alpha$ is characteristic of the specific GPI wave model considered, and result in an underdetermined minimization problem. In order to infer a unique minimizing couple ($h,\nu$), a relationship between effective ice viscosity and thickness has to be empirically formulated through a calibration process.

We perform such calibration thanks to a study conducted in the Odden Ice Tongue, Greenland Sea. The Odden Ice Tongue developed in 1996/1997 and was characterized by massive presence of GPI. The process was extensively documented by SAR imagery, and GPI thickness maps obtained from a specifically developed model were available^30^. Both in the case of the Keller’s and the CP model, calibration produces an effective viscosity which is a monotonically increasing function of the ice thickness.

The proposed calibration technique is eventually tested on four different SAR images gathered by Sentinel-1A and COSMO-SkyMed, representing case studies for the Arctic and for the Antarctic MIZ, respectively. The SAR retrieved GPI thicknesses result in good agreement with the concurrent SMOS measurements and the available local samplings.

## Results

### Empirical constitutive equation for GPI viscosity

The analysis of a set of wave buoy data gathered in the advancing MIZ of the Weddell Sea covered by GPI clearly revealed that the GPI wave attenuation rates scale with the “equivalent solid ice thickness”^21^

3 $$\begin{aligned} h=C_{gr}h_{gr}+C_ph_p, \end{aligned}$$

where *C* and *h* are respectively the concentration and thickness of grease (*gr*) and pancake (*p*) ice. The relation is physically sound, as it states a proportionality between wave decay and ice volume per unit area of the region of the sea traveled by the waves. We point out that the effective ice thickness is the quantity most relevant to the overall changes in ice volume, and is used in numerical dynamic-thermodynamic sea ice models^31^. Hereafter we will refer to the GPI thickness as the effective ice thickness of the GPI layer.

As discussed in the “Methods” section (see Eqs. (21), (22) and (25)), minimization of the cost function $\Psi$ does not fix uniquely the parameters *h* and $\nu$ in the given SAR image; instead, a power law expression is obtained:

4 $$\begin{aligned} \nu =\beta h^\alpha , \end{aligned}$$

where $\alpha$ is specific of the GPI model utilized:

5 $$\begin{aligned} \alpha _{CP}=3, \qquad \alpha _K=-1, \end{aligned}$$

respectively, for the CP and the Keller’s model^25^. On the contrary, the parameter $\beta$ appears to be strongly dependent on the specific SAR image portion examined, and therefore on the physical properties of the sea ice layer and of the wave spectrum in it. Since the cost function in Eq. (2) is integrated over wave numbers, Eq. (4) can depend on wave properties only in integrated form. Moreover, for small amplitude waves, nonlinear dependence of the stress on the wave strain, which would lead to an implicit dependence of $\nu$ on wave properties, can be ruled out. We therefore disregard the dependence of $\nu$ on wave properties and write

6 $$\begin{aligned} \beta =\beta (h)\ \Rightarrow \ \nu =\nu (h). \end{aligned}$$

The underlying hypothesis, which we are going to verify shortly, is that an important component of the GPI’s viscosity variability can be explained through *h* dependence of $\nu$. This would allow one to refine SAR inversion schemes such as the ones by De Carolis (2001 and 2003)^17,18^. From a broader perspective, this suggests that if one uses a viscous layer model to describe wave propagation in a GPI field, a viscosity dependent on the ice thickness is required to reproduce the attenuation data. We consider this an important contribution to the understanding of sea ice rheology, which is an open question for the forecasting/hindcasting of waves in and near the MIZ^32^.

In order to determine the functional form of $\beta$, an alternative representation of the GPI thickness distribution for a given SAR acquisition is required. A possibility is offered by the salt-flux model described in^30^, developed specifically to describe the formation, transport and desalinization of GPI in the Odden region of the Greenland Sea, and carefully validated with in situ measurements. Indeed, an oceanographic campaign was carried out into the Odden from March 3 to March 13 1997 on the R/V Jan Mayen^30^. The Odden locations visited during the cruise operations were imaged by the SAR onboard the ERS2 satellite. In particular, a couple of ERS2 SAR images gathered on March 9, 1997, in coincidence with the acquisition of a Datawell directional wave buoy at (73N, 1W)^14^, and on March 11, 1997, centered at $73.24^{\circ }$ N, $9.11^{\circ }$ W, were considered, respectively.

The equivalent solid ice thicknesses, predicted by the salt-flux model for the areas imaged by the concurrent ERS2 SAR images, were computed according to (3). Comparison of the salt-flux predictions by the model with *in* *situ* ice samplings collected in coincidence with SAR data takes, yields, in the two dates selected, values of the equivalent ice thickness *h* in the range 4-9 cm, with relative error $\Delta h / h\simeq 0.26$.

**Figure 1:**
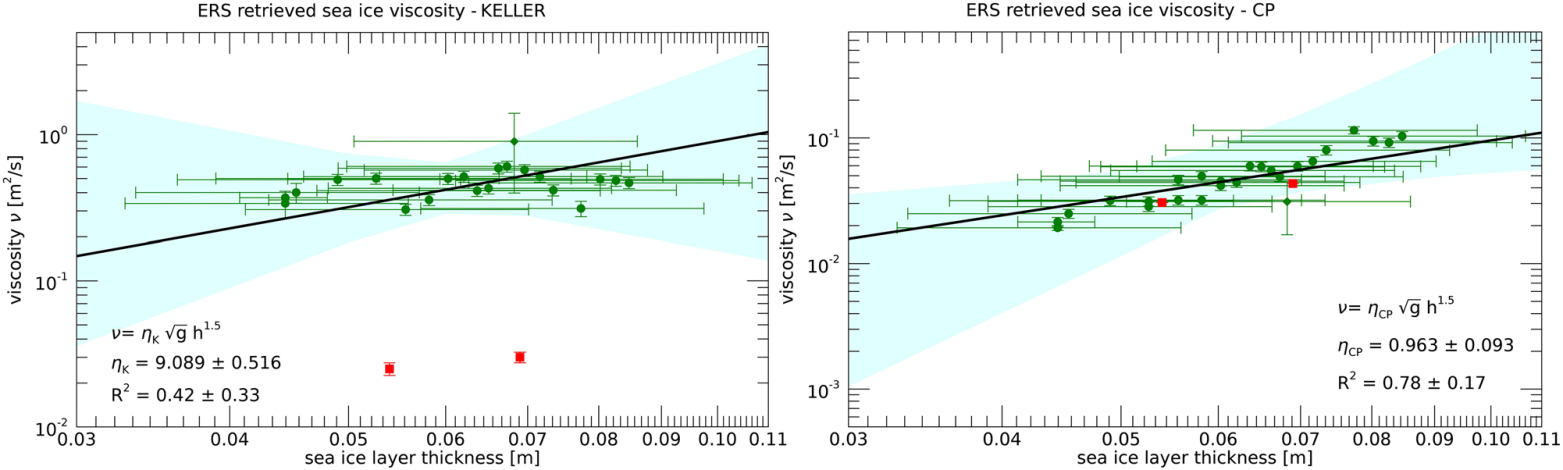
GPI viscosity versus equivalent thickness obtained by running Keller’s model (left panel) and CP model (right panel), for the calibration instances selected from the ERS2 SAR images gathered in the Odden region on 9 March and 11 March 1997, respectively. Viscosity values are given by the absolute minimum of the SAR cost function corresponding to the equivalent ice thicknesses predicted by the ice salt-flux ice model^30^. The two red points represent the pure grease ice viscosity grown in laboratory^27^. Cyan areas represent the regions of 95% confidence level for the best fit line obtained as Bayesian linear regression^33^.

A total of 24 windows at increasing distance from the ice edge were selected for application of the SAR inversion scheme, to sample the overall GPI thickness spatial variability predicted by the salt-flux model. Figure 1 shows the calibration data obtained for both Keller’s and CP models.

**Figure 2:**
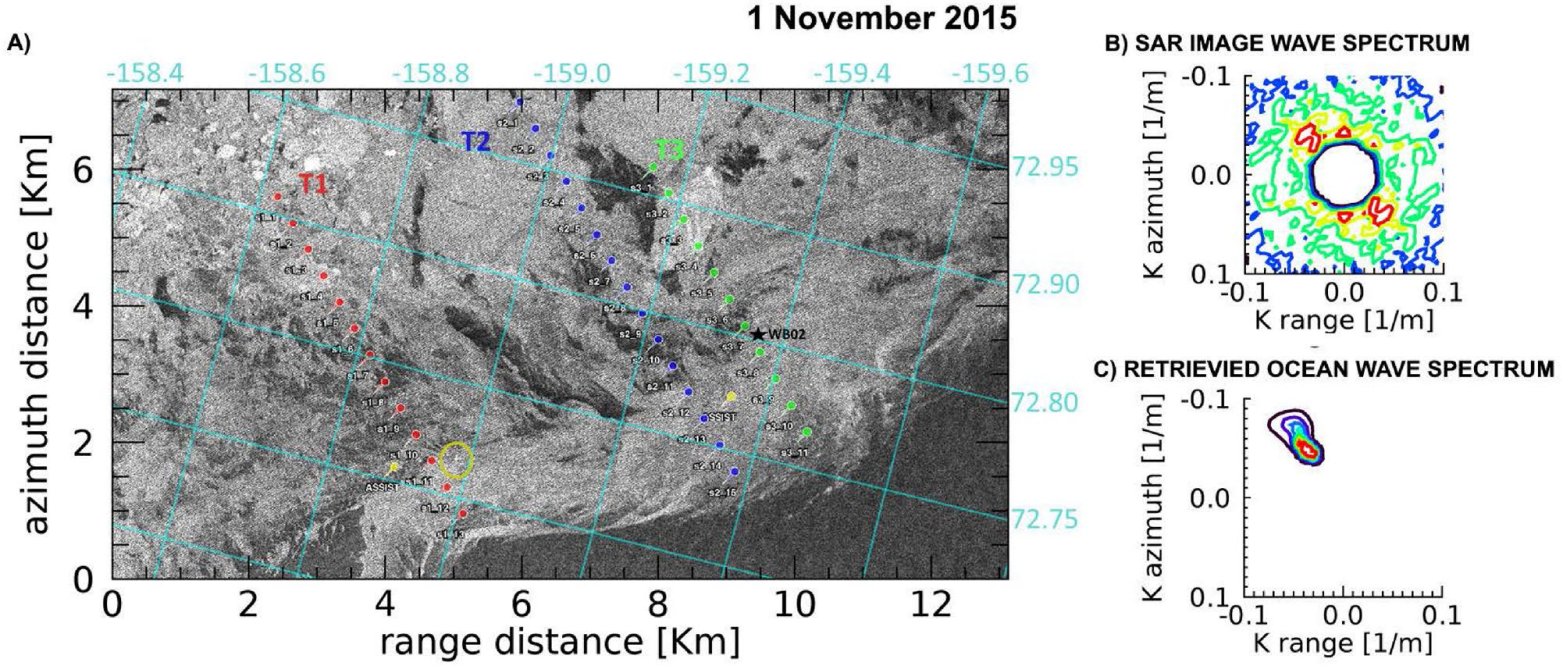
(**A**) Portion of the Sentinel-1A (S1A) IW-mode SAR image acquired on 1 November 2015 at 17:23 UTC in the Beaufort Sea. Representation is in the ground range/azimuth SAR acquisition reference frame. Superimposed is the lat/lon grid. T1 (red), T2 (blue), and T3 (green) are the three transects selected for ice thickness estimation around the corresponding points. The yellow pins indicate the location of ice measurements made according to the ASSIST protocol within ±1 hour the SAR image data take; the black star is the position of the wave buoy WB02 deployed during the field operations and operated to provide the wave field at 17:30 UTC. The bright cross encircled by the yellow ring is the imaged R/V Sikuliaq. (**B**) Observed SAR image wavenumber spectrum in open sea at 72.68$^{\circ }$ N 159.20$^{\circ }$ W. (**C**) Corresponding retrieved ocean wavenumber directional spectrum.

GPI viscosity values were estimated after minimization of the SAR cost function, by imposing the GPI equivalent thicknesses predicted by the salt-flux model. Figure 2 shows the viscosity values obtained for the Keller’s and the CP model. A Bayesian regression scheme^33^ was adopted to account for the uncertainties in the ice viscosity and thickness, in the assumption of a linear relation between ice viscosity and ice thickness on a log-log scale. Figure 1 shows the data used for the fitting procedure to determine the function $\beta (h)$. A coefficient of determination equal to $R^2=0.42$ for the Keller’s model and $R^2=0.78$ for the CP model, respectively, was found. The cyan areas in Fig. 1 represent the region where the straight line is bounded within the 95% confidence level of the linear fit.

The data in Fig. 1 do not allow us to pin down a precise form of constitutive relation $\nu =\nu (h)$. We have chosen to give special weight to the laboratory data in^34^, red points in Fig. 1, which suggest us the empirical law

7 $$\begin{aligned} \nu =\eta g^{1/2} h^{3/2}. \end{aligned}$$

The dimensionless constant $\eta$ may still depend on internal properties of the GPI such as the frazil and pancake size, and the water viscosity. Constant $\eta$ would describe a situation in which the ice response to the waves only depends on characteristic length *h* and characteristic time $(h/g)^{1/2}$, with Eq. (7) providing the only dimensionally consistent expression for the viscosity dependent on the two parameters. We point out that Eq. (7) is conceptually different from Eq. (4). Indeed Eq. (7) represents a constitutive relation for the GPI, while Eq. (4) comes from the viscous model structure and does not carry any information on the ice rheology.

Substituting Eq. (7) into Eqs. (4) and (5), we find in the two cases of the Keller’s and the CP model:

8 $$\begin{aligned} \beta _K=\eta _K g^{1/2} h^{5/2}, \qquad \qquad \beta _{CP}=\eta _{CP} g^{1/2} h^{-3/2}. \end{aligned}$$

The dimensionless coefficient $\eta$ can be evaluated by fitting the Odden data in Fig. 1:

9 $$\begin{aligned} \eta _K=9.089\pm 0.516, \qquad \qquad \eta _{CP}=0.963\pm 0.093 . \end{aligned}$$

The smallness of the uncertainty in $\eta _K$ and $\eta _{CP}$ is an indication that the constitutive relation Eq. (7) provides a physically reasonable description of the rheology of the ice layer. The difference between $\eta _K$ and $\eta _{CP}$, however, is striking. The most natural explanation for this phenomenon is that the viscosity in the Keller model refers to the whole GPI mixture, while in the CP model it refers properly to grease ice layer. In fact, the CP model provides GPI viscosities which are consistent with laboratory measurements^27^, while in the case of the Keller’s model the GPI viscosity is about one order of magnitude higher than the laboratory measurements. We think that such higher viscosity, required to fit the data with the Keller’s model, reflects the contribution of pancakes to the overall ice viscosity. Once the value of $\beta$ from the cost function of the actual SAR inverted tile is available, the average ice thickness of the GPI region travelled by the wave system can be computed from Eq. (9) into Eq. (8). The result is

10 $$\begin{aligned} h=(0.414\pm 0.009)g^{-1/5}\beta _K^{2/5} \end{aligned}$$

for the Keller’s model, and

11 $$\begin{aligned} h=(0.975\pm 0.064)g^{1/3}\beta _{CP}^{-2/3} \end{aligned}$$

for the CP model, respectively. The uncertainty of $\beta$ in Eqs. (10) and (11) is assumed negligible compared to that of $\eta$.

### Examples of SAR inferred GPI thicknesses

Equations (10) and (11) are imposed in the SAR-wave inversion procedure that is applied to map the GPI thicknesses from a Sentinel-1A (S1A) SAR image gathered in the Beaufort Sea, and from three COSMO-SkyMed (CSK) images collected in the Weddell Sea, Antarctica, respectively. All details of SAR images are listed in Table 1.

**Table 1:** Characteristics of the Sentinel-1A and COSMO-SkyMed SAR images selected for the application the SAR inversion procedure to infer GPI thickness. Results of the best fit SAR image spectra at the peak location along with the retrieved ocean wave spectra parameters in open sea are also reported.

Satellite					Observed SAR spectral parameters @ peak location			Retrieved SAR spectral parameters @ peak location			Retrieved ocean wave spectrum parameters		
Date/ Time	Sensor	Band/Pol	Orbit/ look	Pixel size (m)	Value (arb. unit)	Wavelength (m)	Dir. (deg)	Value (arb. unit)	Wavelength (m)	Dir. (deg)	Wavelength (m)	Dir. (deg)	Hs (m)
11/1/2015 17:23	S1A	C/HH	DESC/ RIGHT	10	13	113	225	39	118	214	118	214	1.45
3/21/2019 17:16	CSK1	X/VV	DESC/ RIGHT	15	27	100	133	25	108	106	128	274	2.00
3/22/2019 17:10	CSK4	X/VV	DESC/ RIGHT	15	24	110	114	25	121	108	147	274	2.11
3/30/2019 10:00	CSK4	X/VV	ASC/ RIGHT	15	32	141	234	26	141	234	168	232	1.90

#### Arctic-Beaufort Sea

On 1 November 2015, an IW-mode S1A SAR image was collected in the advancing MIZ of the Beaufort Sea (Fig. 2). At the time of SAR acquisition, the R/V Sikuliaq was in operation to carry out a field cruise as part of a large collaborative program to study the coupled air-ice-ocean-wave processes occurring in the Arctic during the autumn ice advance^9^.

A sharp ice edge can be detected in the S1A SAR image separating the open sea (bottom part) from the ice field. The region of icefield directly exposed to open sea appears to be mainly composed of GPI. The hourly visual observations conducted aboard the R/V Sikuliaq using the ASSIST protocol (http://www.iarc.uaf.edu/icewatch) recognized three ice types, namely, young grey ice, pancakes and first year ice, along with an estimation of the primary thickness and partial concentrations for each ice type. Occasional ship-side retrieval of frazil ice and pancakes samples was also carried out as a concurrent measurement, and a number of directional wave buoys were deployed.

At the time of SAR acquisition, a SWIFT wave buoy was floating in open sea around the ice edge, close to the ship, where an incoming wave spectrum was measured with wave height $H_S\simeq 1.2$ m. Figure 2, panel B, shows the two-dimensional SAR image wavenumber spectrum observed by S1A in a $512\times 512$ pixels open sea area, centered at (72.68N, 159.20W), close to the ice edge and near to the SWIFT buoy location; the corresponding two-dimensional directional ocean wave spectrum, shown in the panel C, is obtained using the SWIFT buoy wave spectrum as first guess input^12^. The SAR inversion procedure returns a consistent wave directional spectrum, with a slightly higher value of wave height $H_S\simeq 1.5$ m (Table 1).

**Figure 3:**
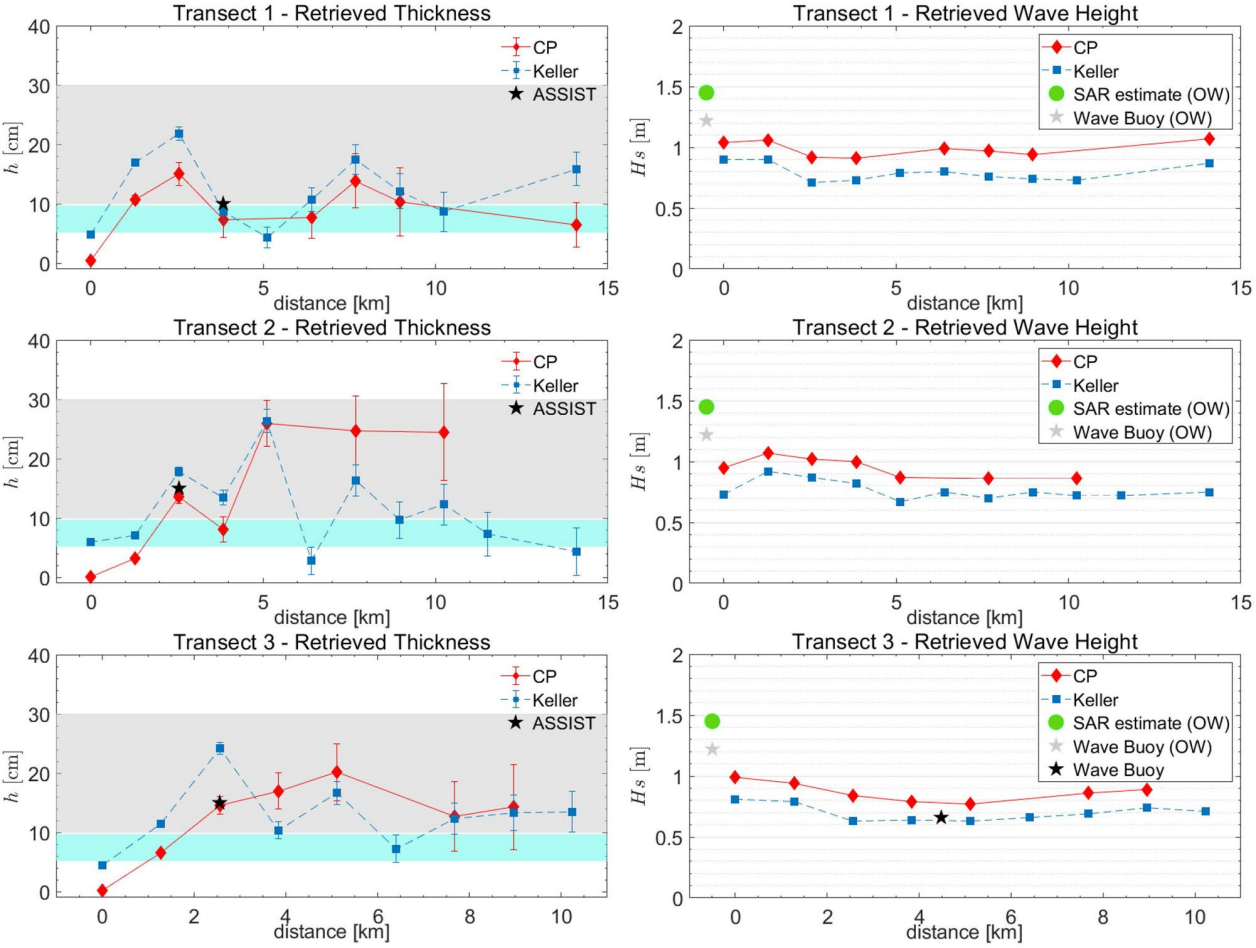
Estimated sea ice thicknesses (left column) and significant wave heights (right column) over the three transects selected on the S1A SAR image using the Keller’s (blue dots) and CP model (red dots), respectively. (left column) The cyan band represents the variability of the SMOS thicknesses in the area covered by the transects. The grey band represents the range of thicknesses of the sea ice cover using the ASSIST protocol on 1 November. The black “star” symbol represents the primary thickness estimate using the ASSIST protocol at the location of the corresponding SAR window within one hour the SAR acquisition. (right column) The green circle indicates the significant wave height $H_s$ estimated from SAR in open water; the grey “star” is the concurrent measurement of $H_s$ in open water from wave buoy S15, the black “star” is $H_s$ measured by wave buoy WB02 in the ice field. Wave buoy names are as indicated in the field campaign reports^9^. WB02 location is signed by a black star in Fig. 2A.

Following the dominant wave direction, three transects were selected, respectively formed by 13 (transect 1, T1), 15 (T2) and 11 (T3) SAR image tiles of size $256\times 256$ pixels, as shown in Fig. 2. The retrieved ice thicknesses are shown in Fig. 3, along with the range of thicknesses from both the ASSIST protocol observations.

Results were also compared with the sea ice thickness retrieved from the L-band microwave sensor Soil Moisture and Ocean Salinity (SMOS). Daily coverage of the polar regions with a resolution of about 35 km $\times$ 35 km are indeed inferred through an empirical method^35^ from SMOS acquisitions. It is worth to note that SMOS sea ice thicknesses (cyan band in Fig. 3) underestimate the primary sea ice thicknesses from ASSIST protocol (grey band in Figure 3).

Thicknesses from SAR retrievals using CP and Keller’s models are all within the SMOS and ASSIST range of thicknesses and show a consistent behaviour running over the ice field depth. The overall trend shows increasing thicknesses going from the ice edge up to values close to 25 cm.

In transect T1 a good agreement between CP and Keller results is observed. At the fourth location, close to the ship, a thickness of $7.3\pm 2.9$ cm from CP model and $8.7\pm 1.5$ cm from Keller’s model are estimated, which are consistent with the 10 cm of primary thickness from ASSIST estimation as well as with the average thickness $9.6\pm 3.8$ cm obtained from pancake measurements collected in six recovery locations^20^.

In transect T2, thicknesses retrieved with both CP and Keller’s models agree for the first 5 km inside the ice fields (windows 1–5). A good correspondence between ASSIST thickness (15 cm) and SAR retrievals (CP: $13.6\pm 1.2$ cm; Keller: $17.8\pm 0.8$ cm), at the third location, is again observed. From window 6 onward, the thicknesses inferred with the two models show different trends. This could be explained by inspecting the SAR image in Fig. 2. In correspondence to window 6, a dark zone is indeed observed, which is consistent with grease ice feature, possibly mixed with water, in the absence of pancakes. In such area wind can easily transfer energy to waves, altering the attenuation trend by the GPI, measured up to window 5.

In this regard, we point out that the SAR inversion procedure compares the open water wave spectrum with the wave spectrum observed at the specific window in sea ice. What the SAR procedure actually returns is therefore the average $h^*_n$ of the effective thickness *h* from the open sea to the given window,

12 $$\begin{aligned} h^*_n=\frac{1}{n}\sum _{i=1}^nh_i. \end{aligned}$$

In order to find the GPI thickness specific only for the window *n*, the contribution from the previous windows has then to be removed as follows,

13 $$\begin{aligned} h_n=nh^*_n-(n-1)h^*_{n-1}. \end{aligned}$$

Negative values for $h_n$ can result. In this case, the data are not reported, as it happens for CP thickness estimation of window 6 belonging to T2. Moreover, note that the CP model specifically represents a systems in which objects, identified as pancakes or small ice floes, float upon a viscous layer, i.e. grease ice. A negative $h_i$ may therefore indicate an absence of pancakes in the considered location, which could lead to wave build-up from wind action.

The two models return thickness values in the following windows of T2 that vary somewhat from window to window, even though they remain in the range defined by the SMOS and ASSIST estimates. This difference in thicknesses could reveal changes of the ice cover, which cannot longer be handled by the CP model.

Finally, as for T1, in transect T3, CP and Keller’s model infer comparable values of the thickness. Also in the third location, there is good agreement between ASSIST thickness (15 cm) and CP thickness ($14.6\pm 1.5$ cm) from SAR, but a slight disagreement with Keller’s thickness, which resulted as high as $24.2\pm 1.0$ cm. Regarding the SAR retrieved wave heights in sea ice, Fig. 3 shows consistent values for CP and Keller’s model. In particular, transect T3 intercepted the wave buoy WB02 about between window 7 and 8, as depicted in Fig. 2. WB02 measured a wave field of $H_s\approx 0.66$ m. It is worth to note that the SAR analysis reported wave heights ranging from 0.63 m at window 7 for the Keller’s model to 0.79 m at window 8 for the CP model, thus demonstrating the effectiveness of the SAR inversion procedure in sea ice.

#### Antarctica-Weddell Sea

In the context of the Year of Polar Prediction (YOPP) initiative of the World Meteorological Program^36^, a survey of the advancing ice edge of the Weddell Sea was performed during March 2019 by exploiting the imaging capability of the four SAR satellites forming the COSMO-SkyMed (CSK) constellation. We have applied the SAR-wave inversion technique to the SAR WIDEREGION images acquired on 21, 22 and 30 March 2019, where at least one CSK SAR satellite was able to image the sea ice edge.

An open ocean wave field of $H_S\simeq 2.0$ m carrying dominant wavelengths ranging from $\lambda \simeq 128$ m to $\lambda \simeq 168$ m is observed to cross the GPI fields in the three dates. The associated wave spectra in open ocean are obtained from SAR inversion using the closest 2D WAM spectrum^37^ available for each date. Details of SAR images characteristics and retrieved wave fields are listed in Table 1.

**Figure 4:**
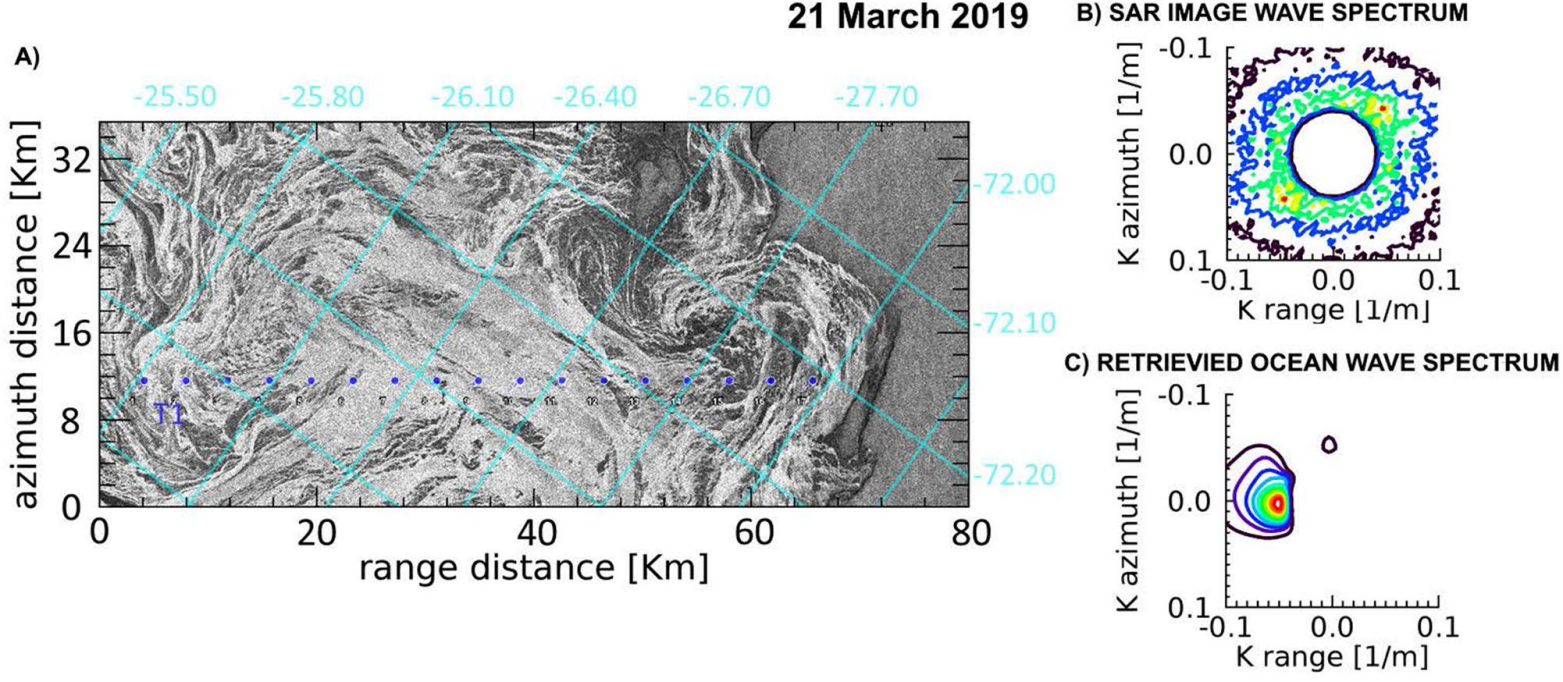
(**A**) Portion of the COSMO-SkyMed Wide Region mode SAR image acquired in the Weddell Sea on 21 March 2019, represented in the geometry of the SAR acquisition reference frame. Superimposed are the lat/lon grids. The aligned blue dots represent the locations along the transect T1 selected for ice thickness estimation. (**B**) Observed SAR image wavenumber spectrum in open sea at (72.00 S, 27.15 W). C) Corresponding retrieved wavenumber directional spectrum in open ocean.

**Figure 5:**
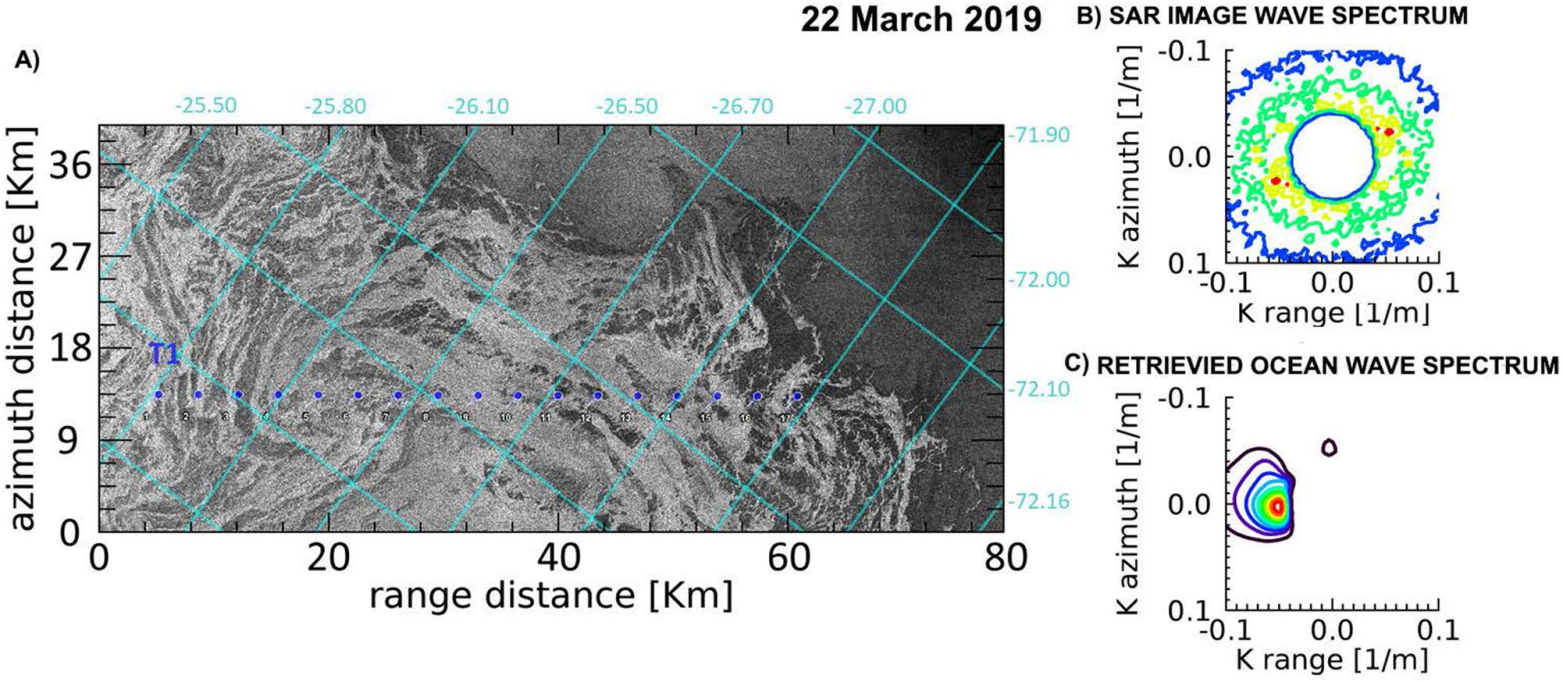
(**A**) Portion of the COSMO-SkyMed Wide Region mode SAR image acquired in the Weddell Sea on 22 March 2019, represented in the geometry of the SAR acquisition reference frame. Superimposed are the lat/lon grids. The aligned blue dots represent the locations along the transect T1 selected for ice thickness estimation. (**B**) Observed SAR image wavenumber spectrum in open sea at (72.00 S, 25.65 W). (**C**) Corresponding retrieved wavenumber directional spectrum in open ocean.

**Figure 6:**
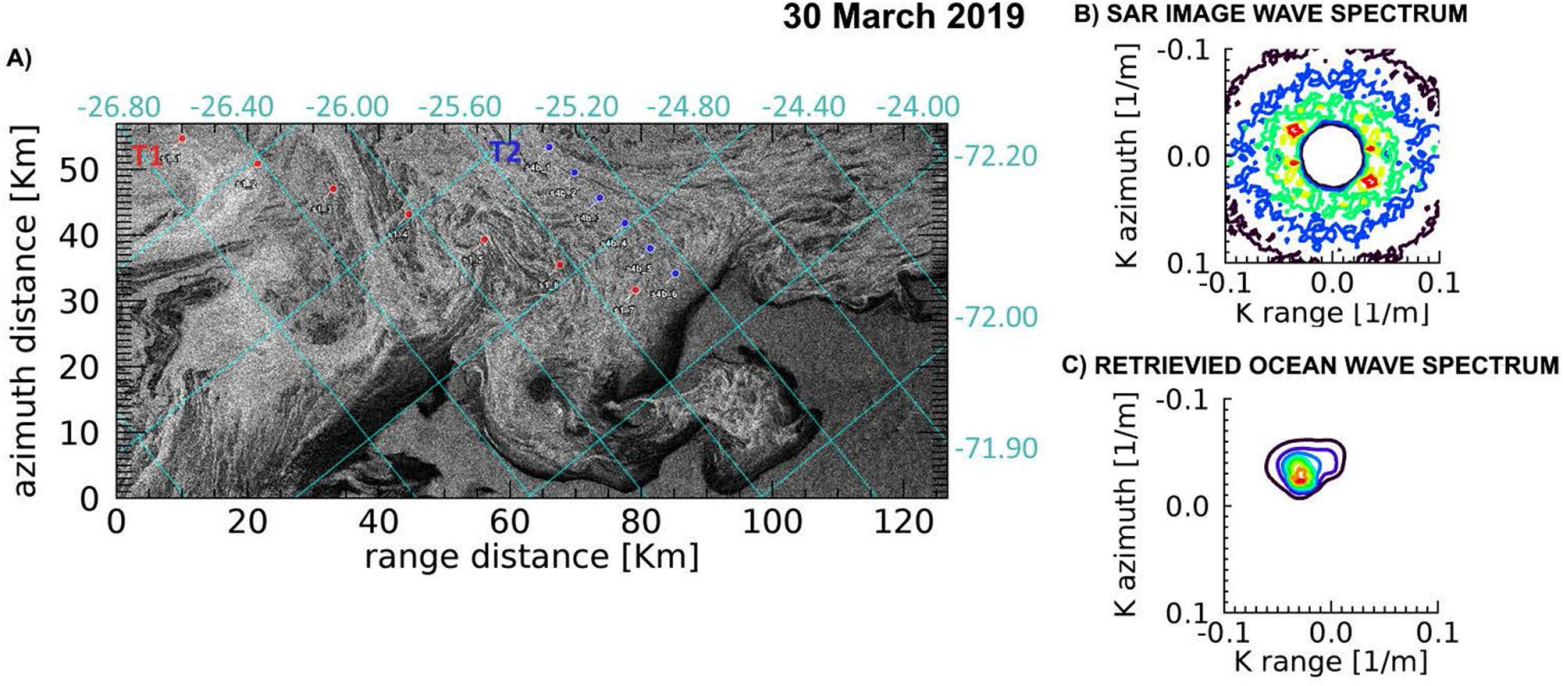
(**A**) Portion of the COSMO-SkyMed Wide Region mode SAR image acquired in the Weddell Sea on 30 March 2019, represented in the geometry of the SAR acquisition reference frame. Superimposed are the lat/lon grids. The aligned red (blue) dots represent the locations along the transect T1 (T2) selected for ice thickness estimation. (**B**) Observed SAR image wavenumber spectra in open sea at (71.95 S, 25.92 W). (**C**) Corresponding retrieved wavenumber directional spectrum in open ocean.

**Figure 7:**
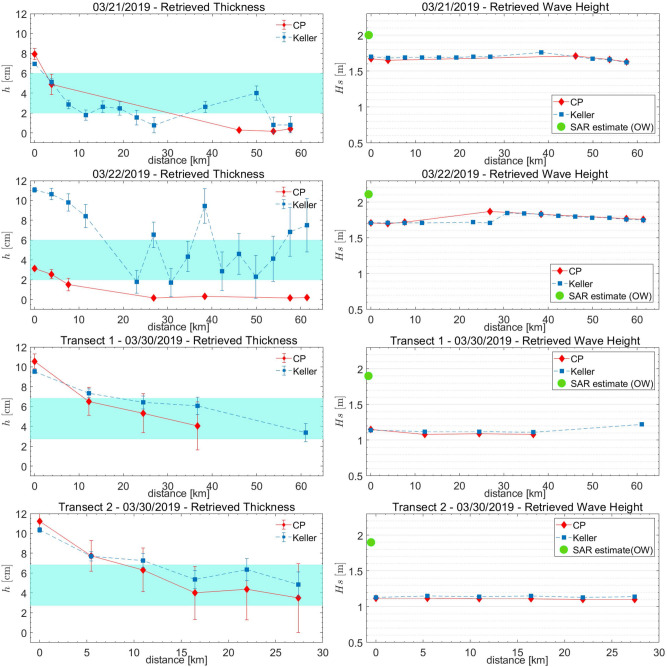
As for Figure 3 but relevant to the transects selected on the CSK SAR images acquired in March 2019.

A common feature of the three SAR images is represented by the dark areas adjacent to the ice edge at direct contact with the open sea, which reveal the presence of bands of pure frazil/grease ice, from which pancakes originate. The SAR images taken on 21 and 22 March almost overlapped the same area of the Weddell Sea and were able to image the outermost part of the developing GPI field; on 30 March, a wider area was imaged by the SAR instrument, which includes the region imaged on 21 and 22 March, thus showing an expansion of the GPI field. Figures 4, 5 and 6 show the interfaces separating open sea with the developed GPI fields imaged by the CSK SAR images where the SAR-wave inversion procedure was applied. As for Fig. 2, transects are selected approximately following the propagation of the dominant wave. For each location of the transects the estimated thicknesses and the significant wave heights are plotted in Fig. 7 according to the distances inside the ice field. The SMOS thicknesses over the area covered by the transects are also reported as band of variability.

As a general comment, smaller values of the thickness, much closer to the SMOS estimations, resulted from the SAR inversion, compared to the Beaufort Sea case study. In the region closer to the ice edge the inferred GPI thickness is about 3 cm higher the extremal SMOS value, with a trend to decrease when running towards the inside of the icefield, in contrast to the case study in the Arctic. The phenomenon could be caused by the action of the wave field that tends to compact the GPI in the region immediately surrounding the ice edge.

Figures 4 and 5 show a very dynamic ice field with many darker areas of pure grease ice, and possibly water, embedded in the GPI environment. As occurred in transect T2 in Fig. 3, also in this case the CP model often appears unable to achieve thickness retrieval. It is worthwhile to note that for the 21st of March a general trend of decreasing thickness is obtained using both models, with comparable values of *h*, when available. Similarly, a general agreement between the inferred thicknesses is observed for the two transects of the 30th of March.

## Discussion

A new technique aimed at the estimation of GPI thickness from the inversion of SAR image wave spectra is proposed. The intrinsic undetermination of any SAR inversion technique based on a viscous layer model is resolved with an empirically determined constitutive relationship between the viscosity and the thickness of the GPI layer, $\nu \propto h^{3/2}$.

A similar power-law relation, $\nu \propto h^2$, was proposed in^38^ on dimensional grounds, based on the hypothesis that $\nu$ only depends on the wave frequency and the ice thickness. We point out that in our case *h* is an effective thickness, proportional through Eq. (3) to the ice volume fraction. The ice volume fraction is expected to increase with *h* under the effect of the buoyancy pressure^39^, which is itself proportional to the effective ice thickness *h*, $P_s\sim \Delta \rho hg$, where $\Delta \rho$ is the mass density gap between ice and liquid water. This gives a physical content to Eq. (7), which goes well beyond the level of a dimensional relation. The microscopic justification of Eq. (7), however, remains elusive, buried in the dependence of the dimensionless parameter $\eta$ in Eq. (7) on internal GPI properties, such as the geometry and size of frazil crystals and pancakes, and the water viscosity $\nu _w$. We point out that any dependence of $\eta$ on $\nu _w$, for dimensional consistency, would require dependence of $\eta$ on an additional time scale, which would require in turn dependence of $\eta$ on *g* or other mechanical parameters, possibly associated with the stress in the ice matrix. In all cases, a picture of the GPI more akin to a brittle solid or a granular medium, than to a fluid suspension^40^, is suggested.

The prefactor in the constitutive equation is determined through a calibration procedure for two different viscous layer models of wave propagation in GPI: the CP and the Keller’s model. To achieve the task, external thickness information provided by the salt-flux model related to the 1997 Odden Ice Tongue^30^ are used as reference data.

The GPI viscosity-thickness relationship obtained for the CP model is comparable with the one found for grease ice grown in wave tank at comparable thicknesses^27^. This is an encouraging result, as the CP model assumes that viscous effects on wave propagation are due to grease ice only. On the other hand, the Keller’s model envisions a layer of grease ice in which pancakes and small floes are suspended. Therefore, the resulting effective viscosity is determined by all types of ice composing the ice layer. A physical interpretation for Eq. (9) is possible following Mooney^41^. Indicating with $\phi$ the volume fraction of the pancakes (suspensions) and with $\phi _c=\pi /6 \approx 0.52$ its value in the case of maximally packed spheres on the sites of cubic lattice, the effective viscosity of the mixture, $\nu$, should obey the following equation:

14 $$\begin{aligned} \ln {\frac{\nu }{\nu _{\mathrm{grease}}}}=\frac{2.5\phi }{1-\phi /\phi _c} \end{aligned}$$

where for $\nu _{\mathrm{grease}}$ is taken the viscosity calibrated for CP. The values obtained for $\eta _{\mathrm{K}}$ and $\eta _{\mathrm{CP}}$ in Eq. (9) are compatible with a layer in which the volume fraction of the pancakes is about $\phi =0.33$. This value of concentration is physically sound and consistent with field measurements reported for the Weddell Sea in Antarctica^8^.

The application of the two calibrated viscosities to different SAR images reveals that the inferred thicknesses obtained by the two models are generally in good agreement, and agree with the other estimates of GPI thickness available. The CP model seems to work better in real GPI fields, when both grease and pancake ice are present; the Keller’s model seems more robust and to work well also in pancake free situations.

## Methods

### The SAR-wave inversion procedure

The approach considers the ocean waves generated in open sea that cross the ice edge and finally propagate in the icefield. It is assumed that the wave spectral features are modified by the GPI cover through viscous interactions modeled either by the CP or Keller’s model. A SAR cost function is defined to measure the distance between the observed SAR image spectrum and the one simulated as a function of the wave models’ parameterization, i.e., ice thickness and viscosity.

#### SAR inversion in open ocean

The first step consists of the estimation of the directional open ocean wave spectrum at the boundary of the icefield from the available SAR image. The corresponding wavenumber SAR image spectrum is estimated from a tile, typically $512 \times 512$ pixels in range and azimuth. If the single look complex SAR image product is available, the SAR image cross-spectrum is calculated starting from the temporal separation between SAR looks, $\tau$. SAR cross spectra significantly reduce the speckle noise level while preserving the spectral shape, and provides information about the wave propagation direction^28^. The simulated SAR image spectrum is computed using the closed form expression of the non-linear ocean-to-SAR spectral form described in Hasselmann and Hasselmann (1991)^12^ and later modified for the SAR image cross-spectral transform^28^. For $\tau =0$ the SAR image cross-spectrum becomes a real-valued quantity and reduces to the standard multilooked SAR image spectrum. According to the procedure proposed by Mastenbroek and De Valk (2000)^42^, the wind-generated ocean spectrum is first estimated. The parametric representation given by Donelan et al. (1985)^43^ is assumed, which depends on the inverse age of the dominant wave, $\Omega = U_{10}/c_p$ , where $U_{10}$ is the 10 m asl wind speed and $C_p$ is the phase velocity of the dominant wave, and the wind vector as well. The latter can be taken either from in situ measurements, if available, or SAR-estimated using the wind vector from a numerical weather prediction model, i.e. ECMWF, as first guess^44^. Then, the residual wave spectrum is estimated by assuming a parametric representation of directional swells according to the JONSWAP-Glenn spectral shape coupled with a directional spreading function of the Mitsuyasu type, properly extended for swell propagation^45^. This choice accounts for non-linearity in SAR imaging features induced by high swells that can occur in the polar seas. Thus, the implemented SAR retrieval of swells adopted a non-linear retrieval scheme which minimizes a cost function with the truncated Newton method implemented in IDL©with respect to the following seven parameters: the dominant wind wavenumber vector; the dominant swell wavenumber vector; the swell wave height; the shape parameters and the directional spread of the swell distribution. The Hasselmann and Hasselmann (1991)^12^ inversion method is applied in alternative, whenever the directional wave buoy data is available.

#### SAR inversion in the GPI field

A series of adjacent SAR tiles of $256\times 256$ pixels centered at the position $\mathbf {x}$ in the SAR reference frame and running from the ice edge along a straight line parallel to the direction of the incoming dominant wave are selected through the GPI field. For each of them, the SAR image (cross-)spectrum is computed. For the SAR images analyzed in this paper, the pixel sizes are 12.5 m for ERS2, 10 m for Sentinel-1A and 15 m for COSMO-SkyMed SAR images. Thus, the spatial resolution of the final GPI thickness and viscosity estimates is of the order of $\approx 10\,\mathrm{km}^2$.

It is assumed that the GPI cover alters the incoming ocean wave spectrum ${\tilde{S}}_w(\mathbf{k})$ as described in Eq. (1). For each window located at position $\mathbf {x}$ into the SAR image, the SAR inversion scheme computes the waves-in-ice spectrum that minimizes the cost function in Eq. (2). The integral definition of the cost function guarantees that all the relevant contributions from the ocean spectrum are gathered through the corresponding SAR image spectrum. After the ocean wave spectrum has been modified according to Eq. (1) through the selected wave model, the simulated SAR image spectrum is computed according to Hasselmann and Hasselmann^12^ and Engen and Johnsen^28^ for $\tau \ne 0$. It is worth noting that application of the SAR inversion procedure requires the following conditions to occur: (i) SAR imaging captures both open ocean and GPI, (ii) the wavefield is suitable for SAR observation, and (iii) it is going toward the GPI. As the methodology requires accurate estimation of wave attenuations to work properly, the incoming waves’ directions and the indentations of the ice edge as seen by the SAR image are accounted for to allow accurate computation of the distances traveled by the waves from the open sea to the point of observation.

### The Keller’s and CP viscous wave propagation models

The two wave propagation models we consider in our analysis assume that sea ice can be treated as a viscous medium. In the presence of gravity waves, viscosity in the ice layer produces a finite normal stress on the ice-free water underneath, thereby modifying the wave dispersion relation. The main difference between the Keller’s and the CP model resides in the way they treat the contribution from pancakes. The Keller’s model implicitly treats GPI as homogeneous medium; the CP model makes the hypothesis that pancakes lie on top of the grease ice layer. The pancake layer is supposed infinitely thin in such a way that its only contribution to the wave dynamics is to generate a finite tangential stress at the top of the grease ice layer. Such tangential stress is obviously absent in the case of the Keller’s model, where the top of the ice layer corresponds to the interface with the atmosphere. In both models the normal stress at the top of the ice layer is zero.

Both models assume a linear wave dynamics. In this regard, although there is evidence for a global increase of typical ocean wave amplitudes^2^, possibly related to global climate change, a linear wave assumption still holds in our analysis. Indeed in all cases here considered, the significant wave height is two orders of magnitude smaller than the peak wavelength (see Table 1).

The tangential stress at the top of the ice layer is controlled in the CP model by a parameter which allows to interpolate between a pancake free situation (corresponding to the Keller model) and a situation in which the pancakes are so closely packed to form an inextensible layer.

The latter regime is the one which concerns us; field measurements by^46^ support the modelling assumption. In this regime both the Keller’s and the CP model are described by just two parameters: the thickness *h* of the ice layer, and an effective ice viscosity $\nu$ which in the case of the CP model refers just to grease ice and in the case of the Keller’s model refers to the whole GPI mixture. It is convenient to introduce the following dimensionless parameters:

15 $$\begin{aligned} {\hat{\nu }}=\frac{k_{\infty }^{3/2}}{g^{1/2}}\nu ,\qquad \psi =\frac{k_{\infty }^{1/4}g^{1/4}}{\nu ^{1/2}}h, \end{aligned}$$

where $k_\infty =\omega ^2/\mathrm{g}$ represents the wave number in open ocean and $g=9.8 {\mathrm{m/s}}^2$ the gravitational acceleration. We can express the two parameters ${\hat{\nu }}$ and $\psi$ in terms of the wavenumber *k*, the ice thickness *h* and the viscous boundary layer depth $\lambda _\alpha =(\omega /\nu )^{1/2}$, as ${\hat{\nu }}=(k\lambda _\alpha )^{1/2}$ and $\psi =h/\lambda _\alpha$. We thus see that small ${\hat{\nu }}$ and $\psi$ correspond to a small ice contribution to the wave dynamics; in our analysis ${\hat{\nu }}$ ranges from $10^{-5}$ to $10^{-1}$ and $\psi$ ranges from $10^{-2}$ to $10^{-1}$.

For small ${\hat{\nu }}$ and $\psi$, the dispersion relations for the CP and the Keller’s model take a very simple form,

16 $$\begin{aligned} \mathrm{CP:}&\qquad&k\approx k_{\infty }+{\hat{\rho }}hk_{\infty }^2+\frac{{\mathrm{i}}}{\hat{\rho }}g^{1/2}k_{\infty }^{5/2}{3}\frac{h^3}{\nu }, \end{aligned}$$

17 $$\begin{aligned}{\mathrm{Keller:}}&\qquad&k\approx k_{\infty }+4{\mathrm{i}}\frac{{\hat{\rho }}k_{\infty }^{7/2}}{g^{1/2}}h \nu . \end{aligned}$$

where ${\hat{\rho }}\approx 0.92$ is the ratio between the GPI density and the ocean water density. We can identify in Eq. (16) a dispersion contribution that reproduces the result by the mass-loading model^10^. The wave attenuation is the imaginary part of the wavenumber vector, $q=\mathfrak {I}(k)$, that has in the two cases the simple power law form $q\propto h^3/\nu$ (CP model) and $q\propto h\nu$ (Keller’s model).

The *h*-dependence of $\nu$ in Eqs. (16) and (17) can be made explicit by applying the calibration results in Eqs. (7) and (9):

18 $$\begin{aligned} {\mathrm{CP:}}&\qquad&k\approx k_{\infty }+{\hat{\rho }}hk_{\infty }^2+\frac{{\mathrm{i}}{\hat{\rho }}}{3\eta _{CP}} k_{\infty }^{5/2}h^{3/2}, \end{aligned}$$

19 $$\begin{aligned} {\mathrm{Keller:}}&\qquad&k\approx k_{\infty }+4{\mathrm{i}}{\hat{\rho }}\eta _{K}k_{\infty }^{7/2}h^{5/2} . \end{aligned}$$

#### Geometric structure of the cost function minima

For small *h* the asymptotic relations Eqs. (16) and (17) can be rewritten as

20 $$\begin{aligned} q(h,\nu ;\omega )=A(h,\nu )B(\omega ), \end{aligned}$$

where for the Keller’s model

21 $$\begin{aligned} A(h,\nu )=h \nu ,\qquad B(\omega )= 4\frac{{\hat{\rho }}k_{\infty }^{7/2}(\omega )}{g^{1/2}}, \end{aligned}$$

and for the CP model

22 $$\begin{aligned} A(h,\nu )=\frac{h^3}{\nu },\qquad B(\omega )=\frac{{\hat{\rho }}g^{1/2}k_{\infty }^{5/2}(\omega )}{3}. \end{aligned}$$

Substituting Eq. (20) into Eq. (1) yields

23 $$\begin{aligned} S_i(\mathbf{x},\mathbf{k}_\infty ;h,\nu )={\tilde{S}}_w(\mathbf{k}_\infty )\exp [-2A(h,\nu )B(\omega (\mathbf{k}_\infty ))\Delta (\mathbf{e}_\mathbf{k},\mathbf{x})], \end{aligned}$$

which tells us that the cost function $\Psi$ in Eq. (2) depends on the variables *h* and $\nu$ only through $A(h,\nu )$:

24 $$\begin{aligned} \Psi (\tau ,\mathbf{x};h,\nu )=\Psi (\tau ,\mathbf{x};A(h,\nu )). \end{aligned}$$

The minimum of the cost function is determined by imposing

$$\begin{aligned} 0=\nabla _{\nu ,h}\Psi =\frac{\partial \Psi }{\partial A}\nabla _{\nu ,h}A, \end{aligned}$$

whose solution can be written in the form

25 $$\begin{aligned} A_{min}(\nu ,h)=F(\tau ,\mathbf{x}). \end{aligned}$$

Combining Eqs. (21), (22) and (25) then yields Eqs. (4) and (5).

**Figure 8:**
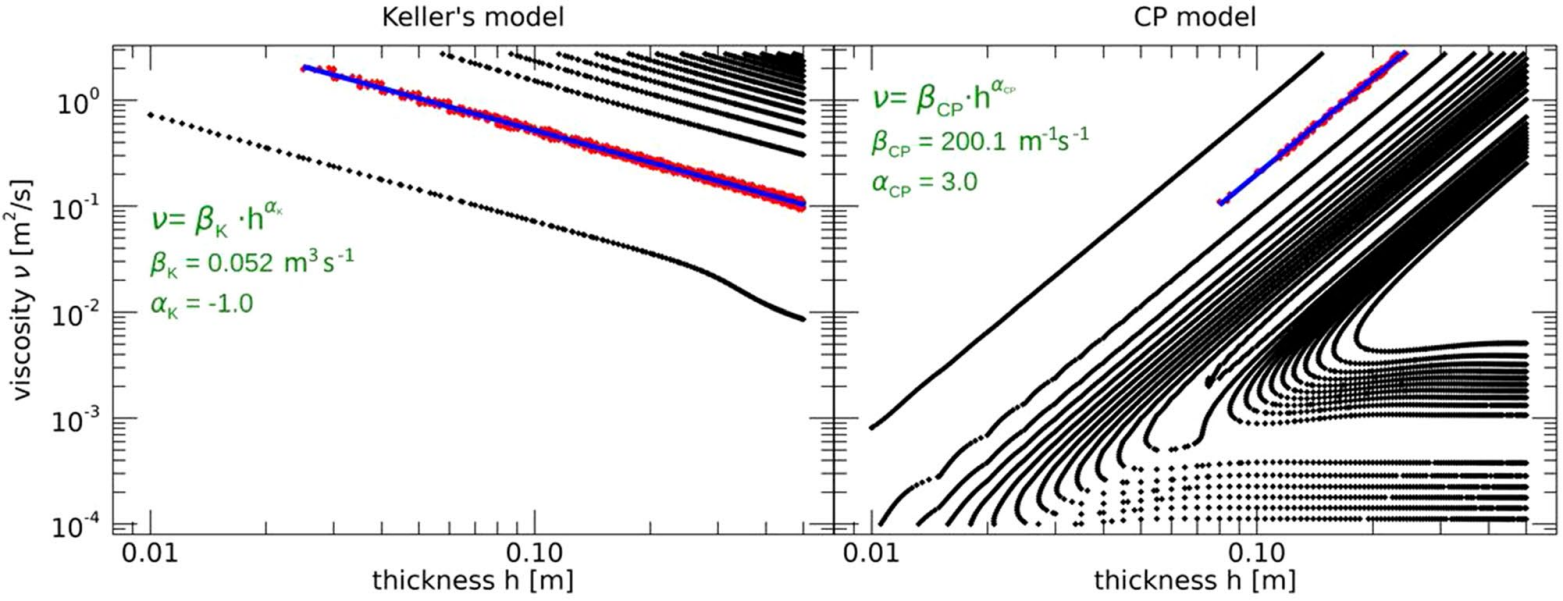
Exemplary contour plots of the cost functions obtained from SAR inversion in GPI field for the Keller’s model (left panel) and the CP model (right panel). Red points mark those locations $(h,\nu )$ where the cost function values are within 1% of the corresponding absolute minimum. This example corresponds to the window number 13 selected on the ERS2 SAR image of the 9 March 1997 and is representative for any cases considered in this paper.

Although the above procedure can only be justified in the limit of small *h*, we verify that the result in Eqs. (4) and (5) continues to hold for realistic values of *h*. The contour lines in Fig. 8 are obtained by simulating the SAR image wave spectrum with the full wave dispersion relation. As this trend is common to all the examined cases, we assume that Eq. (4) always holds.
